# Development and application of an electronic treatment register: a system for enumerating populations and monitoring treatment during mass drug administration

**DOI:** 10.1080/16549716.2020.1785146

**Published:** 2020-07-15

**Authors:** William E. Oswald, David S. Kennedy, Jasmine Farzana, Saravanakumar Puthupalayam Kaliappan, Eloic Atindegla, Parfait Houngbégnon, Alvin Chisambi, Stefan Witek-McManus, Sean R. Galagan, Mira Emmanuel-Fabula, Marie-Claire Gwayi-Chore, Hugo Legge, Elodie Yard, Khumbo Kalua, Moudachirou Ibikounlé, Sitara Swarna Rao Ajjampur, Arianna Rubin Means, Kristjana H. Ásbjörnsdóttir, Katherine E. Halliday, Judd L. Walson

**Affiliations:** a DeWorm3, Division of Life Sciences, Natural History Museum, London, UK; b Faculty of Infectious and Tropical Diseases, London School of Hygiene & Tropical Medicine, London, UK; c The Wellcome Trust Research Laboratory, Division of Gastrointestinal Sciences, Christian Medical College, Vellore, India; d Institut de Recherche Clinique du Bénin (IRCB), Calavi, Benin; e Blantyre Institute for Community Outreach, Lions Sight First Eye Hospital, Blantyre, Malawi; f Department of Global Health, University of Washington, Seattle, WA, USA; g Département de Zoologie, Faculté des Sciences et Techniques, Université d’Abomey-Calavi, Cotonou, Benin; h Department of Epidemiology, University of Washington, Seattle, WA, USA

**Keywords:** Neglected Tropical Diseases, soil-transmitted helminths, deworming, electronic data collection, monitoring

## Abstract

We developed an electronic treatment register for the DeWorm3 Project, a cluster-randomised, controlled trial in Benin, India, and Malawi testing the feasibility of interrupting transmission of soil-transmitted helminths through community-wide mass drug administration. The electronic treatment register was designed in xlsform, deployed via the SurveyCTO mobile data collection platform, and implemented on smartphones running the Android operating system. The versatile system enables collection of census and treatment status information, facilitates data aggregation and visualisation, and permits real-time feedback loops during implementation of mass drug administration. Here we describe the system’s design and use within the DeWorm3 Project and key features, and by sharing the register here, we hope our readers will further explore its use within their research and disease-control activities.

## Background

Population treatment coverage is a fundamental indicator of mass drug administration (MDA) performance for neglected tropical disease (NTD) programmes [1,2]. Defined as the proportion of the targeted population who swallows the recommended drug or drug combination, treatment coverage is ideally measured at point of delivery, based on directly observed treatment [1,3]. Data collection during MDA routinely uses paper treatment registers or tally-sheets to record and summarise numbers treated by sex and age. These data must ultimately be tabulated and reported at the implementation unit level and then nationally. This approach may lead to compromised data quality due to user error, delayed implementation feedback and reporting, and an increased workload for community drug distributors (CDDs) and health information officers. Critically, systematically missed populations or coverage equity cannot be readily or reliably identified using this approach, despite recognition of the importance of high coverage and compliance in reaching WHO Roadmap targets [2].

Electronic data collection for public health using mobile phones or devices, or mHealth, has increased in recent years, matching availability and functionality of phone and internet technologies. Use of mobile devices for large-scale NTD prevalence surveys has been described [4,5], and, though examples exist of applying mobile technology for monitoring MDA for NTD control [6–9], a recent review highlighted the lack of documentation of mHealth interventions [10].

Here, we describe the creation and application of an innovative electronic data collection and monitoring system for registering individuals and their treatment status during community-wide MDA activities implemented for the DeWorm3 Project [11]. This system can improve quality, accuracy, and comprehensiveness of MDA data, facilitate household targeting, and reduce time needed to access data for implementation monitoring.

The DeWorm3 Project (ClinicalTrials.gov Identifier NCT03014167) is a multi-site cluster-randomised, controlled trial conducted in Benin, India, and Malawi. The project has been described previously, but, briefly, DeWorm3 aims to test feasibility of interrupting transmission of soil-transmitted helminths (STH) through three years of expanded MDA targeting all community members [11]. Study clusters were randomised to receive either the standard of care (current national STH MDA strategy) or intervention. In the trial’s intervention arm, biannual, community-wide MDA with a single dose of albendazole (GlaxoSmithKline) is delivered house-to-house by CDDs targeting approximately 55,000 individuals.

### Development and application of the electronic treatment register for DeWorm3

Drawing from experience from the TUMIKIA Project [12], we designed the electronic treatment register for the DeWorm3 Project to avoid the challenges with paper-based data collection described above, while permitting real- or near real-time implementation and coverage monitoring. At the time of development, we knew of no existing system for mobile data collection during MDA that could be adapted to meet the needs of the project and that would sit alongside the project’s other data collection tools and workflows. Towards this, the register was developed in xlsform (xlsform.org) for use with SurveyCTO (Dobility, Inc; Cambridge, MA, USA) running on Android smartphones.

The register is used by field officers accompanying CDDs as they treat. The development process involved extensive field-based piloting, working with site data teams to optimise and translate the register for use under varied contexts and technological constraints. For example, the Malawi site lacks widespread cellular data access, so data are submitted when field officers are able to access internet hotspots. In contrast, at the India and Benin sites, data are submitted after each household visit. The xlsform and associated files are available at the following link (https://github.com/williameoswald/deworm3_etr), and setup and use protocols are available from the DeWorm3 research toolkit (http://www.nhm.ac.uk/our-science/our-work/sustainability/deworm3/research-tool-kit.html). A functional web version of the register (http://tinyurl.com/y2azojsc) and dashboard (https://tinyurl.com/y3rx3w88) are also available.

**Figure 1.**
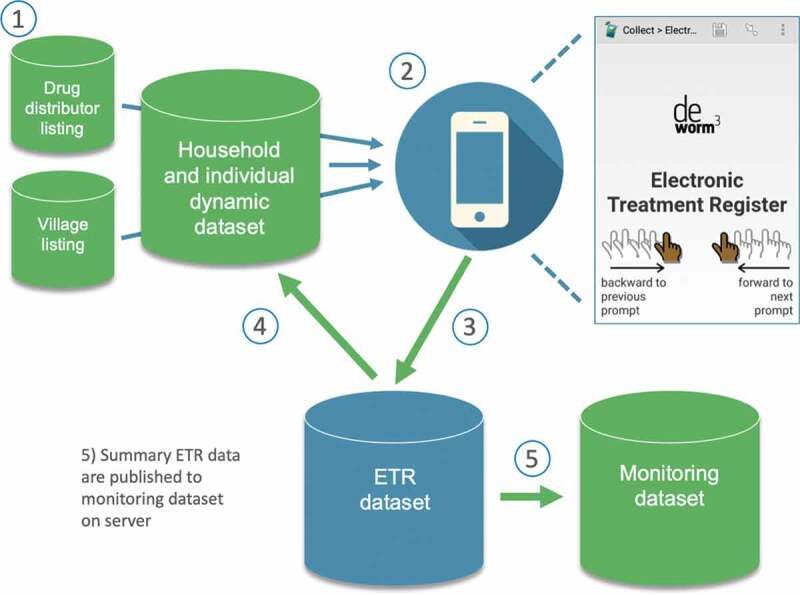
The electronic treatment register (ETR) data system for enumerating populations and registering individual treatment during community-wide mass drug administration.

Trial site data systems are built upon a comprehensive listing of villages, higher administrative units, and an annually updated household census to enumerate residents and collect socioeconomic information and dwelling GPS coordinates. Household information, including each member’s name, age, and sex, is assembled into a dataset that is pre-loaded in the electronic treatment register. Field officers access the register in their local language through the SurveyCTO app and use displayed resident information to record receipt of treatment or reason for non-treatment (Figure 1).

## Results

### Improving data quality with a dynamic treatment register

The dataset attached to the register is dynamic, meaning household, individual, and treatment information is updated on field officers’ smartphones to reflect changes between visits. Beyond the logic checks and range constraints programmed within the electronic treatment register, this dynamic dataset improves data quality. For example, tracking individual presence and treatment between visits avoids duplication of records on paper registers that might occur if households receive multiple visits by different CDDs.

### Provision of a population denominator

The electronic register enables collection of accurate community-level population information, necessary for calculating treatment coverage. DeWorm3 activities benefit from a pre-MDA census, but if there are new household members encountered during a visit, their information and treatment status can be collected and then updated at later visits. In the same way, new households can also be added to the dataset. This dynamic functionality would allow either: (1) a pre-MDA census to first list a community’s households and residents, which is then updated with treatment status during MDA; or (2) resident demographic and treatment information to be collected during MDA, starting from an empty dataset.

### A flexible tool to facilitate treatment

The electronic treatment register facilitates targeting of households, improves comprehensiveness, and reduces workload. Households to be targeted are listed within the register and are then automatically filtered to remove them from this listing once all members have been treated. Using an approach provided by Dobility, Inc., GPS coordinates are displayed in the form as a uniform resource locator (URL) that can then be viewed in Google Maps (Google LLC, Mountain View, CA, USA) to navigate to targeted households. Finally, the register is flexible to treatment delivery location. Field officers choose to record treatment of an entire household (all members) or, after making at least one visit to a household’s dwelling, to record treatment of a single member of that household found elsewhere in the community (e.g. at work).

### Real-time monitoring and reactive decision-making

**Figure 2.**
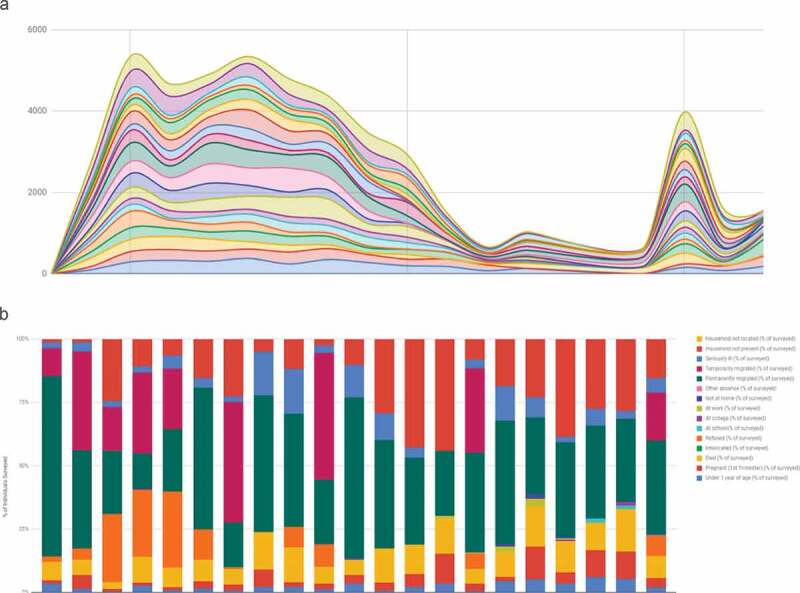
Sample dashboard visualisations representing A) number of treatments delivered per day by cluster and B) frequency of recorded reasons for non-treatment by cluster.

Electronic collection means data are readily available for analysis and visualisation. Household treatment summaries are calculated per visit within the register, removing need for manual calculation. These summaries are submitted as part of the household record to the SurveyCTO server where they are automatically pseudonymised and assembled into a monitoring dataset. This household monitoring dataset is then published from the SurveyCTO server to a dashboard in Google Sheets (Google LLC, Mountain View, CA, USA), where it is further aggregated by cluster, date, and field officer. Access to SurveyCTO and the household level data on Google is restricted to specific users. The dashboard then displays treatment summaries, allowing a wider group of users to monitor progress and coordinate implementation during MDA (Figure 2).

## Discussion

Here we present our electronic treatment register as a data collection system. The backbone of the system is the provided xlsform, and this tool can be modified by anyone with experience authoring such forms in Microsoft Excel (Microsoft Corporation, Redmond, WA, USA). We developed the electronic treatment register to be implemented via SurveyCTO, but the provided xlsform and described process, including data management, aggregation, and visualisation, could potentially be implemented via free data collection and analysis platforms, like Open Data Kit [13] and R (r-project.org). By sharing our tool and a description of our MDA data collection process, we aim to facilitate future data collection activities for researchers and provide an additional tool for potentially strengthening programmatic data collection.

The WHO recently recommended the use of electronic data collection and storage, or ‘digital tracking’ of health information, with decision support in settings where integration of such interventions can be supported by the health system and for tasks within the scope of practice for the health worker [14]. In line with similar recommendations that community health workers could document the services they provide using relevant mobile health solutions [15], the electronic treatment register, though developed for a research project, is simple to use and could incorporate graphics instead of text for users with limited literacy. Coupled with availability of inexpensive smartphones and growing internet access, the register provides a versatile platform to replace paper-based data collection, aggregation, and analysis workflows. Collected data can then be more readily acted upon during implementation, easily integrated within national information system platforms (e.g. DHIS2), and more easily accessed for the completion of preventive chemotherapy donation reporting requirements [16]. Users should, however, consider and comply with local ethical and data protection standards.

## Conclusion

Our electronic treatment register could potentially improve MDA data quality and accuracy and timeliness of feedback and reporting, which in turn may boost coverage. We now encourage readers to further explore and adapt the use of our register within their own research or disease control activities.

## Paper context

Paper-based approaches for recording treatment during mass drug administration (MDA) are widely used but contribute to high implementer workloads and may compromise data quality. Resulting data may not be available in time to act upon to improve coverage during MDA. For the DeWorm3 project, we developed an ‘Electronic Treatment Register’ that we have used to enumerate populations and accurately track treatments provided in real-time, improving timeliness of reporting and strengthening data use towards maximising coverage.
